# Short-term application of diquafosol ophthalmic solution benefits children with dry eye wearing orthokeratology lens

**DOI:** 10.3389/fmed.2023.1130117

**Published:** 2023-07-13

**Authors:** Yuanfang Yang, Qinghui Wu, Yao Tang, Haoran Wu, Zhiwei Luo, Wenyu Gao, Ziqi Hu, Lijun Hou, Min Wang, Zhikuan Yang, Xiaoning Li

**Affiliations:** ^1^Aier School of Ophthalmology, Central South University, Changsha, China; ^2^Aier Institute of Optometry and Vision Science, Changsha, China; ^3^Hunan Province Optometry Engineering and Technology Research Center, Changsha, China; ^4^Hunan Province International Cooperation Base for Optometry Science and Technology, Changsha, China; ^5^Changsha Aier Eye Hospital, Changsha, China; ^6^Shanghai Aier Eye Hospital, Shanghai, China; ^7^Aier College of Ophthalmology and Optometry, Hubei University of Science and Technology, Xianning, China

**Keywords:** diquafosol, ocular surface, discomfort, tear film stability, orthokeratology

## Abstract

**Purpose:**

This aim of this study was to evaluate the effect of 3% Diquafosol Ophthalmic Solution (DQS) on children with dry eye from wearing overnight orthokeratology (OrthoK) lenses.

**Methods:**

Myopic children aged 8–18 years with dry eye syndrome were enrolled in this prospective observational study, and they were grouped according to their OrthoK treatment history for at least 1 year. All participants received DQS 4 times per day for 1 month. The following indicators were measured at baseline 1 month after treatment: the Dry Eye Questionnaire-5 (DEQ-5), non-invasive tear meniscus height (TMH), non-invasive tear film break-up time (first and average, NIBUT-F and NIBUT-A), meibomian gland score (MG score), conjunctival hyperemia redness score (R-scan), and blink pattern analysis.

**Results:**

A total of 104 participants (189 eyes) including 40 OrthoK wearers (72 eyes) and 64 Orthok candidates (117 eyes) completed the study. Of all, after DQS treatment for 1 month, DEQ-5 scores reduced from 5.54 ± 3.25 to 3.85 ± 2.98 (*t* = −3.36, *p* = 0.00). TMH increased from 0.20 ± 0.05 mm to 0.21 ± 0.05 mm (*t* = 2.59, *p* = 0.01), NIBUT-F and NIBUT-A were prolonged from 6.67 ± 4.71 s to 10.32 ± 6.19 s and from 8.86 ± 5.25 s to 13.30 ± 6.03 s (all *p* = 0.00), respectively. R-scan decreased from 0.69 ± 0.28 to 0.50 ± 0.25 (*t* = −9.01, *p* = 0.00). Upper MG scores decreased from 1.04 ± 0.32 to 0.97 ± 0.36 (*t* = −2.14, *p* = 0.03). Lower MG scores, partial blink rate, partial blinks, and total blinks did not change significantly. Both break-up time (BUT) and R-scan improved significantly after DQS treatment for 1 month (all *p* = 0.00) in OrthoK candidates and OrthoK wearers. Among the OrthoK wearers, TMH and dry eye symptoms increased significantly (all *p* = 0.00) but did not increase in OrthoK candidates (*p* > 0.05). There were no adverse events related to DQS.

**Conclusion:**

Diquafosol Ophthalmic Solution was effective for children wearing overnight orthokeratology in relieving dry eye symptoms and improving ocular surface parameters, which may help improve children's OrthoK wearing tolerance and compliance.

## Introduction

The prevalence of myopia in children has increased markedly worldwide, especially in East and Southeast Asia (1, 2). The burden of pathological consequences caused by myopia lead to irreversible blindness, such as myopic maculopathy and high myopia-associated optic neuropathy (3). Orthokeratology (OrthoK) is effective in controlling myopia progression in children (4, 5), which has gained increasingly wide application (6–8). The safety and comfort of OrthoK wearing have become a primary concern and are important for maintaining corneal morphology (9) to ensure the effect and safety of myopia control (10). Contact lens discomfort, especially contact lens-related dry eye (CLADE), is a major cause of the discontinuation of contact lens wear (11). According to the Tear Film and Ocular Surface Society Dry Eye Workshop II, even 33.33% of “healthy” children had a dry eye disease (12). Besides, contact lens wearing is believed to be one of the main causes of dry eye (13). Ocular surface problems of dry eyes in children wearing OrthoK have raised increasing attention from physicians (14).

Studies have highlighted that there is an interplay between tear film stability and effect, that is, the safety of OrthoK wearing. The hydrostatic pressure or negative pressure generated by the accumulation of tears in the reversing arc area of the OrthoK lens forms a strong positive pressure around the base arc area, while the tears in the surrounding arc are continuously sucked into the reversing arc area by a similar siphon effect to supplement the tear loss caused by the positive pressure, forming a circulating system (15). The quantity and the quality of the tear may affect the prescription, effect, and safety of OrthoK fitting and wearing, for example, they affect the measurement of corneal curvature (16), the repeatability of axial measurement (17), and the speed and amplitude of OrthoK in controlling myopia (10). Meanwhile, OrthoK wearing may affect tear stability and ocular surface health. Previous studies have found that OrthoK use may damage the ocular surface in adolescents. Some adolescents experience ocular discomfort symptoms and tear film instability (18), corneal staining (19), or even meibomian gland atrophy (20). Tear-related visual function parameters were correlated with ocular discomfort, while 40% of the patients reported dry eye or itch about 1–2 times per week during OrthoK wear at night (21). Dry eye discomfort symptoms can offset the visual benefits although current studies suggest that satisfaction with OrthoK is positive (22). However, rare studies pay attention to the diagnosis and treatment of dry eye syndrome in children. While previous dry eye treatment options for children are similar to those for adults; the initial treatment consists of artificial tear eye drops and environmental recommendations (23). Owing to the popularization and widespread usage of OrthoK, it is urgent to improve the level of treatment of dry eye in children through OrthoK use.

Diquafosol, a P2Y2 receptor agonist, stimulate both water secretion from conjunctival epithelial cells and mucin secretion from conjunctival goblet cells (24, 25). It can improve tear secretion and prevent corneal epithelial damage in dry eye animal models of rabbits (26) and rats (27). Besides, studies have also shown that diquafosol may induce the release of total cholesterol from rabbit blephomain cells by P2Y2 purine receptor signal transduction. This reveals that diquafosol may have a positive effect on the three-layered tear structure (28). Approximately 3% Diquafosol Ophthalmic Solution (DQS^®^) exhibited effects similar to and superior to those of sodium hyaluronate in the treatment of adults with dry eyes (29–32). Real-world clinical practices also confirmed that the topical application of DQS could be an effective and safe treatment for children with dry eyes (33, 34). For persistent dry eye patients after LASIK (35, 36) and cataract surgery (37, 38), DQS improved tear film stability and dry eye symptoms. In addition, DQS is safe for patients with contact lens-related dry eye, and it also mitigates ocular surface damage and subjective symptoms (39, 40).

As dry eye in children has its own characteristics, we lack relevant information on the treatment and effectiveness of dry eyes in children. Moreover, it is unknown whether the efficacy of DQS treatment for dry eye is associated with OrthoK wearing in children. Thus, we conducted this study to evaluate the efficacy and safety of DQS for OrthoK candidates and OrthoK wearers with dry eyes based on keratograph and LipiView tests.

## Materials and methods

### Study design and participants

This prospective open-label study was conducted at Changsha Aier Eye Hospital between February 2022 and July 2022. All participants were provided with a full explanation of the study and provided their written informed consent. The Institutional Ethics Committee of Changsha Aier Eye Hospital approved the study (approval No.: KYPJ002, 2022). All procedures were conducted following the principles of the Declaration of Helsinki.

The inclusion criteria were as follows: (1) age between 8 years and 18 years; (2) myopia from −1.00 Diopter to −5.50 D, with-the-rule astigmatism of up to −1.75 D or against-the-rule astigmatism of less than −0.75 D with keratometry from 41.00 to 46.00 D; (3) participants were first-time users of OrthoK or had worn OrthoK for at least 1 year; (4) participants with a potential risk of mild-to-moderate dry eye based on TMH <0.20 mm or BUT <10 s from Keratograph 5M examinations adopted by the Dry Eye Workshop (41); and (5) participants cooperated with eye drop usage as required, completed examinations, and went back to the hospital for follow-up examinations within the specified time. The exclusion criteria were as follows: (1) participants with allergic or autoimmune diseases associated with dry eye are not suitable for OrthoK lenses; (2) participants had pathological changes of the lid margin, cornea, uvea, retina, and other systemic diseases that may influence the ocular surface, for example, serious ocular surface disease (e.g., Sjögren syndrome, allergic conjunctivitis, ocular pemphigoid, conjunctivochalasis, conjunctival scarring, and chemical injury); (3) participants had received any dry eye treatment within 14 days before the start date of this study or continued to use other topical ophthalmic solutions that can affect the study results; and (4) participants received other ocular treatments or surgeries.

### Measurement protocol

Participants were divided into two groups depending on their history of wearing OrthoK, namely, the OrthoK wearers group (who had worn OrthoK lenses for at least 1 year) and the OrthoK candidates group (new wearers without a history of using any contact lens). All participants received DQS^®^ (3% Diquafosol Ophthalmic Solution; Santen Pharmaceutical Co. Ltd., Osaka, Japan) 4 times per day for 1 month. The usage frequency of DQS (4 times daily) and follow-up time (1 month) in this study were based on the studies conducted by Hwang et al. (42) and Holland et al. (43). It is more feasible and rigorous to observe its effect and safety on adolescents with 4 times daily for 1 month. We also recorded age, sex, habit outdoors, the screen using time, daily circumstances, and slit-lamp examination. We administered Questionnaire-5 (DEQ-5) and measured non-invasive keratograph tear film break-up time (NIBUT, first and average, BUT-F BUT-A), non-invasive tear meniscus height (TMH), conjunctival hyperemia redness score (R-scan), upper and lower meibomian gland scores (MG scores), and blink pattern. All these dry eye-related indicators were evaluated at baseline and 1 month after DQS treatment. Two skilled physicians performed baseline and follow-up examinations for all participants without awareness of the participants' medication use and OrthoK lens wear history to ensure the reliability of the examination results.

### Subjective symptoms

Subjective symptoms were assessed using Dry Eye Questionnaire-5 (DEQ-5) (44). DEQ-5 was used to compare the dry eye-related symptoms at baseline and 1 month after DQS treatment, and a total score greater than 6 indicated dry eye symptoms. The questionnaire consisted of the following five questions:

Q1: During a typical day in the past month, how often did your eyes feel discomfort?
Q2: When your eyes felt discomfort, how intense was this feeling of discomfort at the end of the day, within 2 h of going to bed?
Q3: During a typical day in the past month, how often did your eyes feel dry?
Q4: When your eyes felt dry, how intense was this feeling of discomfort at the end of the day, within 2 h of going to bed?
Q5: During a typical day in the past month, how often did your eyes feel excessively watery?

### Ocular surface examination

TMH, BUT-F, BUT-A, R-scan, and MG score were conducted with the Keratograph 5M (Oculus GmbH, Wetzlar, Germany) to evaluate the ocular surface status and tear film stability at baseline and 1 month after DQS treatment. Participants were instructed to look straight ahead, and TMH, R-scan, and MG scores were measured in TMH quantitative photography mode, R-scan quantitative photography mode, and MG photography mode, respectively. Meibomian glands loss scores were graded as 0 (no loss), grade 1 (dropout < 1/3), grade 2 (dropout 1/3–2/3), and grade 3 (dropout >2/3). BUT was generated by automatic detection and calculation as follows: (1) BUT-F, the time at which the first distortion in the reflected Placido ring occurred and (2) BUT-A, related to the localized TBUTs and calculated based on the average time of all detected perturbations.

### Blink pattern analysis

Partial blink rate (PBR), partial blinks, and total blinks were recorded using LipiView Ocular Surface Interferometer (Johnson & Johnson, USA). During the examination, the patients were asked to look at the front lightspot to ensure that their pupils were directly in the center of the interferometer camera with natural blinking, and all the measurements were conducted by the same experienced examiner. Participants with an outcome conformance factor of < 0.8 were asked to repeat the measurement.

### Adverse events

At every visit, the occurrence of systemic adverse events was checked. If adverse events were found, the findings were reported.

### Statistical methods

Statistical analysis was performed with IBM SPSS Statistics 26.0 (IBM SPSS, Inc., USA). The continuous measurement data subjected to normal distribution were presented as the mean ± SD, and the enumeration data were presented as the ratio (%). The missing data incurred during examinations in this study were at random, and the proportion was very small ( ≤ 7%); in addition, relevant missing data were not included in the statistics. The chi-squared test was used for baseline enumeration of variables such as sex, parental smoking, daily outdoors (h), and daily electronic screen time (h). Baseline continuous data, such as DEQ-5 scores, TMH, NIBUT, R-scan, MG Scores, PBR, partial blinks, and total blinks, were compared between the two groups using the independent samples *t*-test. We used the paired *t*-test to obtain overall and intergroup differences of continuous data between, before, and after DQS treatment responses throughout the study period. The significance level was set as α = 0.05.

## Results

### Study subjects

A total of 104 participants (189 eyes) completed the Keratograph 5M and LipiView examination at baseline and 1 month after DQS treatment. The OrthoK wearers group consisted of 40 participants (72 eyes), and the OrthoK candidates group consisted of 64 participants (117 eyes). The baseline demographic data are summarized in Table 1. There was no significant difference in sex (*p* = 0.26), parental smoking (*p* = 0.68), daily outdoors (*p* = 0.33), and daily electronic screen time (*p* = 0.62) between OrthoK candidates and OrthoK wearers, except for age (11.49 ± 2.29 vs. 12.58 ± 2.16, *p* = 0.00).

**Table 1:** Baseline demographic data of OrthoK candidates and OrthoK wearers.

	**OrthoK candidates**	**OrthoK wearers**	***t***	***p***
*N*	117	72		
Age	11.49 ± 2.29	12.58 ± 2.16	−3.26	0.00^**^
**Sex**				
Male (%)	57.30%	42.70%		0.26
Female (%)	65.40%	34.60%		
**Parental smoking**				
Yes	56.00%	44.00%		0.68
No	60.50%	39.50%		
Daily outdoors (h)	1.42 ± 0.79	1.24 ± 0.80	0.98	0.33
Daily electronic screen time (h)	1.36 ± 1.13	1.23 ± 1.25	0.49	0.62

** *p* < 0.01.

The baseline characteristics of the ocular evaluation are summarized in Table 2. There was no significant difference in DEQ-5 scores (*p* = 0.06), TMH (*p* = 0.07), BUT-F (*p* = 0.84), BUT-A (*p* = 0.50), and R-scan (*p* = 0.13), Upper MG Scores (p=0.88), and total blinks (*p* = 0.62) between OrthoK candidates and OrthoK wearers at baseline. However, there were significant differences in lower MG scores (1.08 ± 0.40 vs. 1.00 ± 0.00, *p* = 0.04), PBR (0.63 ± 0.35 vs. 0.82 ± 0.28, *p* = 0.00), and partial blinks (4.06 ± 3.26 vs. 5.78 ± 3.78, *p* = 0.00) between OrthoK candidates and OrthoK wearers at baseline.

**Table 2:** Baseline characteristics of dry eye symptoms and ocular surface of OrthoK candidates and OrthoK wearers.

**Baseline**	**OrthoK candidates**	**OrthoK wearers**	***t***	***p***
**Subjective symptoms**				
DEQ-5 scores	4.79 ± 3.36	6.60 ± 2.85	−1.96	0.06
**Ocular surface examination**				
TMH (mm)	0.20 ± 0.06	0.19 ± 0.05	1.83	0.07
BUT-First (s)	6.62 ± 4.61	6.76 ± 4.91	−0.20	0.84
BUT-Avg (s)	8.65 ± 5.24	9.19 ± 5.28	−0.68	0.50
R-scan	0.71 ± 0.27	0.65 ± 0.28	1.51	0.13
Upper MG scores (0–3)	1.03 ± 0.32	1.04 ± 0.31	−0.16	0.88
Lower MG scores (0–3)	1.08 ± 0.40	1.00 ± 0.00	2.09	0.04^*^
Partial blink rate (PBR)	0.63 ± 0.35	0.82 ± 0.26	−4.36	0.00^**^
Number of partial blinks	4.06 ± 3.26	5.78 ± 3.78	−3.20	0.00^**^
Number of total blinks	6.38 ± 3.40	6.64 ± 3.69	−0.50	0.62

* *p* < 0.05 and

** *p* < 0.01.

### Subjective symptoms

**Figure 1:**
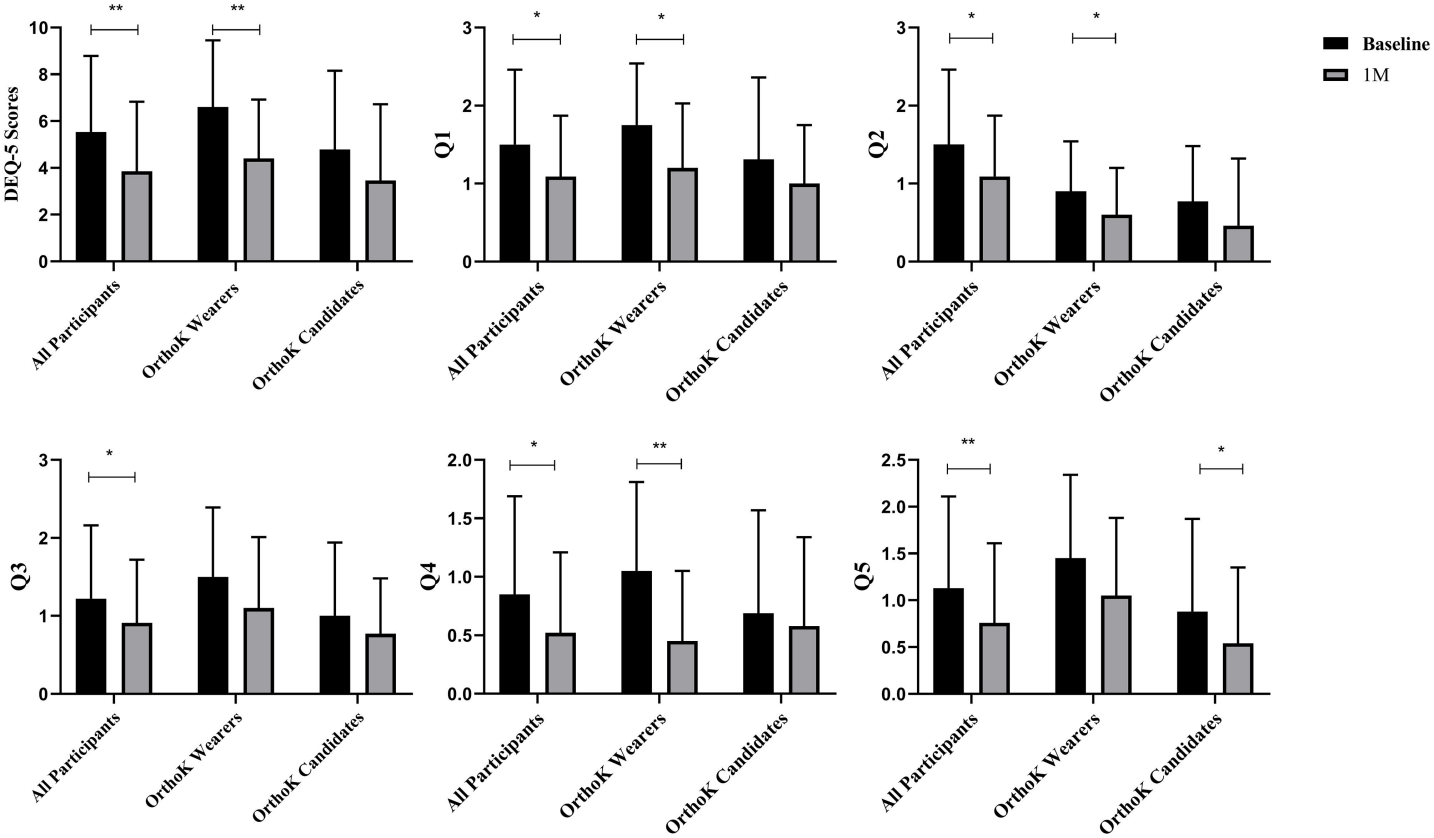
Subjective symptoms: changes in Dry Eye Questionnaire-5 (DEQ-5) scores before and after DQS treatment. Q1: During a typical day in the past month, how often did your eyes feel discomfort? Q2: When your eyes felt discomfort, how intense was this feeling of discomfort at the end of the day, within 2 h of going to bed? Q3: During a typical day in the past month, how often did your eyes feel dry? Q4: When your eyes felt dry, how intense was this feeling of discomfort at the end of the day, within 2 h of going to bed? Q5: During a typical day in the past month, how often did your eyes feel excessively watery? *p < 0.05 and **p < 0.01.

Figure 1 shows the changes in subjective symptoms from baseline to 1 month after DQS treatment. Among all participants, compared with the baseline, the DEQ-5 scores decreased (5.54 ± 3.25 vs. 3.85 ± 2.98, *t* = −3.36, *p* = 0.00). Among OrthoK wearers, the DEQ-5 scores (6.60 ± 2.85 vs. 4.40 ± 2.52, *t* = −4.22, *p* = 0.00), Q1 (1.75 ± 0.79 vs. 1.20 ± 0.83, *t* = −2.98, *p* = 0.01), Q2 (0.90 ± 0.64 vs. 0.60 ± 0.60, *t* = −2.35, *p* = 0.03), and Q4 (1.05 ± 0.76 vs. 0.45 ± 0.60, *t* = −3.27, *p* = 0.00) decreased from baseline to 1 month, whereas among OrthoK candidates, there was a significant decrease only in Q5 (0.88 ± 0.99 vs. 0.54 ± 0.81, *t* = −2.37, *p* = 0.03).

### Ocular surface examination

**Figure 2:**
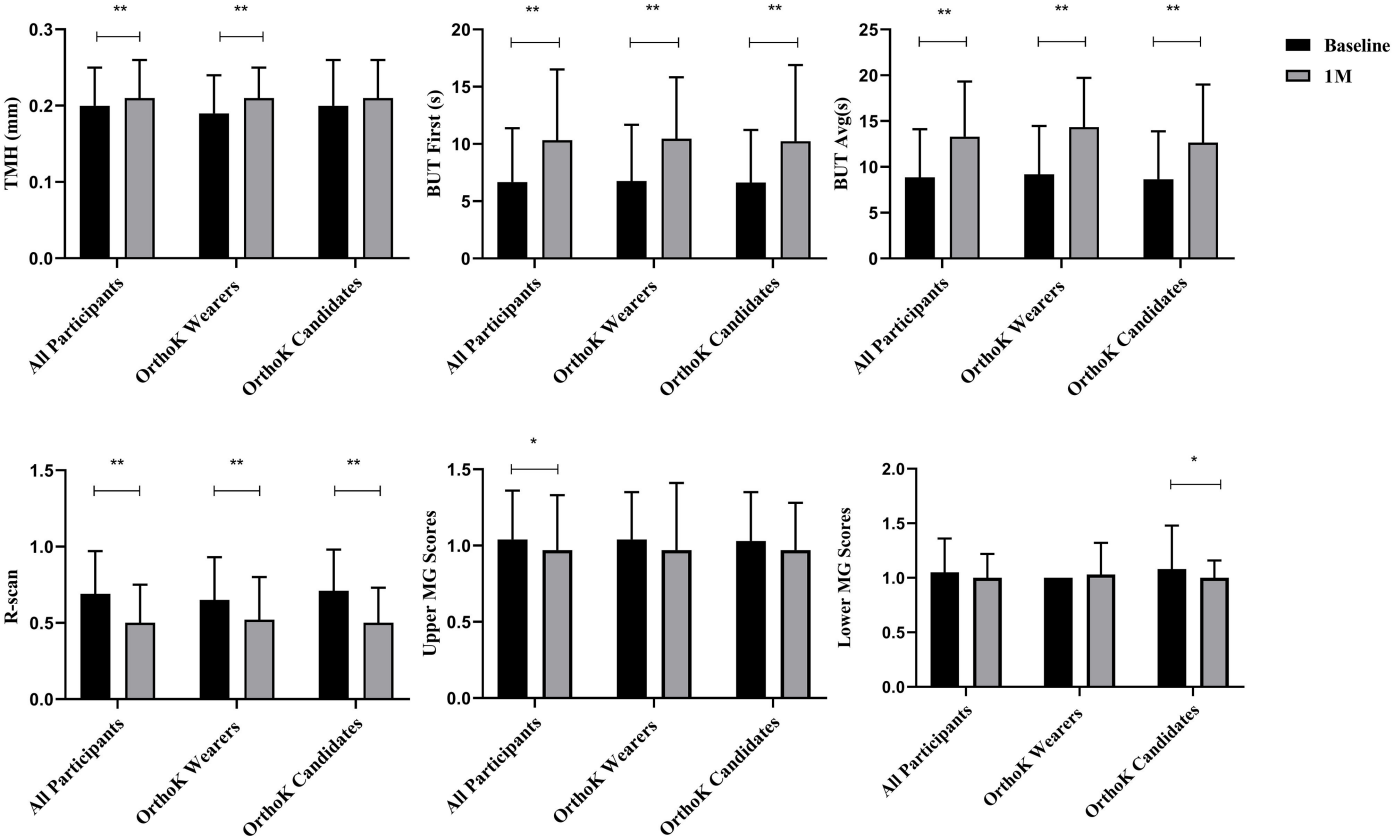
Ocular surface examination: changes in TMH, BUT-F, BUT-A, R-scan, upper MG scores, and lower MG scores before and after DQS treatment in all participants, the OrthoK wearers group, and the OrthoK candidates group. *p < 0.05 and **p < 0.01.

Figure 2 shows the changes in the ocular surface from baseline to 1 month after DQS treatment. Among all participants, compared with the baseline, TMH (0.20 ± 0.05 vs. 0.21 ± 0.05, *t* = 2.59, *p* = 0.01), BUT-F (6.67 ± 4.71 vs. 10.32 ± 6.19, *t* = 7.20, *p* = 0.00), BUT-A (8.86 ± 5.25 vs. 13.30 ± 6.03, *t* = 9.42, *p* = 0.00), R-scan (0.69 ± 0.28 vs. 0.50 ± 0.25, *t* = −9.01, *p* = 0.00), and upper MG scores (1.04 ± 0.32 vs. 0.97 ± 0.36, *t* = −2.14, *p* = 0.03) improved significantly after 1-month DQS treatment. However, lower MG scores did not change significantly (*p* = 0.10). Of OrthoK candidates and OrthoK wearers, compared with the baseline, BUT-F (6.62 ± 4.61 vs. 10.24 ± 6.66, *t* = 5.62, *p* = 0.00 and 6.76 ± 4.91 vs. 10.45 ± 5.38, *t* = 4.46, *p* = 0.00, respectively), BUT-A (8.65 ± 5.24 vs. 12.65 ± 6.33, *t* = 7.02, *p* = 0.00, and 9.19 ± 5.28 vs. 14.34 ± 5.37, *t* = 6.30, *p* = 0.00, respectively), and R-scan (0.71 ± 0.27 vs. 0.50 ± 0.23, *t* = −8.68, *p* = 0.00 and 0.65 ± 0.28 vs. 0.52 ± 0.28, *t* = −3.84, *p* = 0.00, respectively) improved after 1-month DQS treatment; however, Upper MG scores did not change significantly (*p* = 0.11, *p* = 0.17). Among the OrthoK wearers, TMH increased significantly (0.19 ± 0.05 vs. 0.21 ± 0.04, *t* = 3.18, *p* = 0.00), but not in OrthoK candidates (*p* = 0.32). However, among the OrthoK candidates, lower MG scores (1.08 ± 0.40 vs. 1.00 ± 0.16, *t* = −2.41, *p* = 0.02) decreased significantly but not in OrthoK wearers (*p* = 0.42).

### Blink pattern analysis

**Figure 3:**
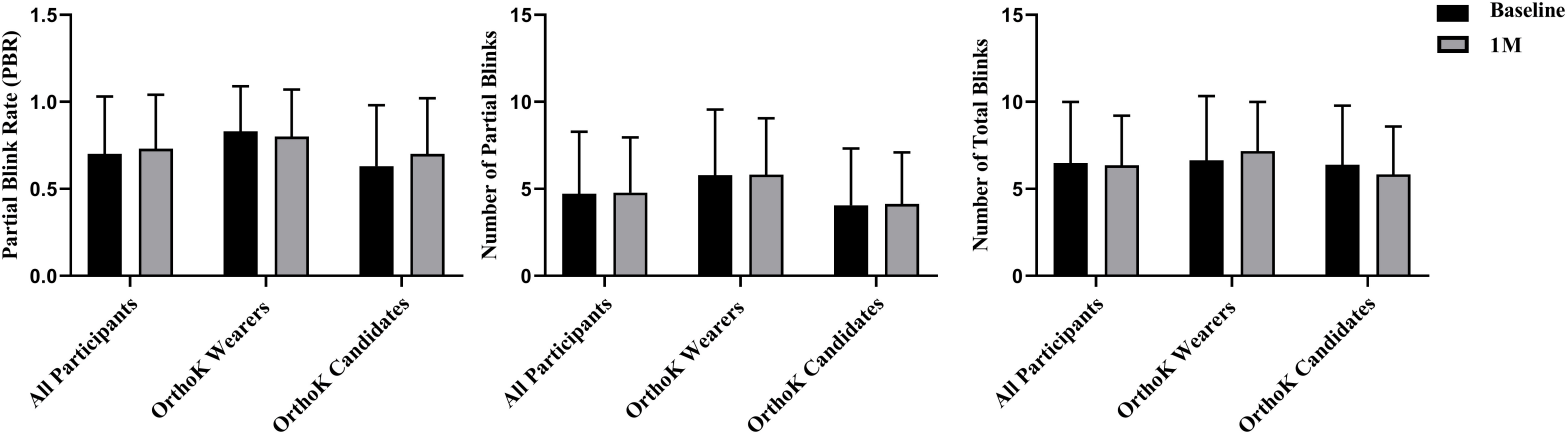
Blink pattern analysis. Changes in PBR, partial blinks, and total blinks before and after DQS use in both groups, the OrthoK wearers group, and OrthoK candidates group.

Figure 3 shows the changes in the blink pattern from baseline to 1 month. Among all participants, compared with baseline, no significant changes were observed in PBR (0.70 ± 0.33 vs. 0.73 ± 0.31, *t* = 1.00, *p* = 0.31), partial blinks (4.72 ± 3.56 vs. 4.78 ± 3.18, *t* = 0.18, *p* = 0.86), and total blinks (6.48 ± 3.51 vs. 6.36 ± 2.85, *t* = −0.42, *p* = 0.68) after DQS treatment. Of OrthoK candidates and OrthoK wearers, no significant changes were observed in PBR (*p* = 0.11 and *p* = 0.42), partial blinks (*p* = 0.88 and *p* = 0.93), and total blinks (*p* = 0.15 and *p* = 0.26) from baseline to 1 month.

### Safety

All participants adhered to the DQS treatment for 1 month, and no adverse reactions or events developed during the observation period.

## Discussion

We investigated the efficacy and safety of DQS on OrthoK lens-related dry eye symptoms and measured the ocular surface parameters in children with OrthoK wearing history or candidates for OrthoK. Owing to the poor coordination with the examination of children, we used non-invasive Keratograph 5M (45) and LipiView (46) in this study to obtain objective parameters. Besides, we balanced the effect of time spent outdoors, electronic screen use, and parental smoking that might affect tear quality (14).

At baseline, DEQ-5, TMH, BUT, R-scan, and total blinks were similar between OrthoK wearers and OrthoK candidates; however, the DEQ-5 score and TMH were slightly worse in OrthoK wearers, suggesting that wearing OrthoK lenses may impact a wearer's subjective symptoms as well as tear quantity. We also found that lower MG scores were higher in OrthoK candidates than OrthoK wearers at baseline, this may be due to meibomian gland growth with aging (47) considering the age difference between Orthok candidates and wearers. Similarly, OrthoK wearers had higher PBR and partial blinks than OrthoK candidates, but similar total blinks at baseline. To our knowledge, total blinks can promote reconstruction and generation of the lipid layer to sustain tear film stability of the ocular surface; however, partial blinking is detrimental (48, 49). OrthoK influences the tear film stability, which in turn alters the children's blinking habits. According to the results confirmed by Hui et al. (50), ocular surface and meibomian gland function did not change significantly although wearing OrthoK lenses may have aggravated dry eye symptoms. Children's dry eye subjective symptoms may precede objective examinations, and it is essential to explore the effectiveness and safety of treatment of the ocular surface in children with OrthoK. This may also indicate that, when OrthoK children have ocular surface examinations abnormality, there may already exist symptoms that require a physician's attention.

In this study, through subjective symptoms analysis, we found that the frequency of eye discomfort, the intensity of eye discomfort, and the intensity of dry eye sensation were diminished more significantly in OrthoK wearers than in OrthoK candidates. OrthoK wearers had significant improvement in TMH than OrthoK candidates. Ocular surface subjective parameters were significantly improved in children with topical DQS in this study, which is consistent with the results of Kojima et al. (51) for adult dry eye treatment. Besides, our study revealed that OrthoK wearers are more sensitive to DQS in promoting tear secretion and relieving contact lens-related discomfort than those without a contact lens-wearing history. We considered that OrthoK may disturb tear film stability and that DQS just compensates quite well for the negative influences. Of course, further long-term observation of DQS's sustained effect on OrthoK wearers is needed. TMH, NIBUT, DEQ-5 scores, R-scan, and upper MG scores of all participants also improved significantly after 1-month DQS treatment. As previous studies showed that DQS significantly improved BUT and subjective symptoms in soft contact lens-related dry eye (40) and also can stabilize tear film stability (31, 52). This study demonstrates the efficacy and safety of DQS on children and children with or without wearing OrthoK. In our study, we have proven that DQS could improve subjective and objective parameters in children with dry eyes. We believe that, although this is a short-term observation, it may inspire physicians to make clinical decisions when dealing with dry eye children before and during wearing OrthoK.

Our study showed no significant difference in blink patterns, including PBR, partial blinks, and total blinks, after 1-month DQS treatment. However, we ascertained that the blink pattern was similar or slightly improved after DQS use for a month. However, in the baseline, the blink pattern was worse in the OrthoK wearers than in the OrthoK candidates. However, other investigators found that the topical application of DQS alleviated meibomian gland dysfunction (53, 54). We hypothesize that the blink pattern did not change due to the increase in tear film stabilization and meibomian gland dysfunction through using DQS. Blink patterns, partial blinks in specific, were important in assessing mild-to-moderate dry eyes and associate well with other ocular surface parameters (49). We also hypothesize that alleviation of dry eye symptoms is first manifested in tear film stability and meibomian gland function. While blink pattern improvement required additional long-term medication, some studies have reported that contact lens wearers with dry eyes benefit from increased blinking frequency, which may help them to reduce dry eye symptoms and to improve the ocular surface environment (55, 56). In this study, there was no change in blink patterns in children with or without a history of OrthoK after 1-month of DQS treatment, which demonstrated that DQS may have a stabilizing effect on blink patterns in children in short-term use.

This study inevitably has some limitations. First, it was a single-center, single-arm research project that lacks a control population, and a large sample randomized controlled trial is recommended. Second, this study was only a short-term observation, and long-term prospective studies need to be further explored. Meanwhile, further studies are needed to evaluate tear composition and distribution and how these factors are influenced by tear film stability and meibomian gland status.

## Conclusion

Diquafosol Ophthalmic Solution was effective and safe for children wearing overnight orthokeratology in alleviating dry eye symptoms and ocular surface parameters, which may help improve children's OrthoK wearing tolerance and compliance.

## Data availability statement

The raw data supporting the conclusions of this article will be made available by the authors, without undue reservation.

## Ethics statement

The studies involving human participants were reviewed and approved by the Institutional Ethics Committee of Changsha Aier Eye Hospital. All procedures were conducted following the principles of the Declaration of Helsinki. Written informed consent to participate in this study was provided by the participants' legal guardian/next of kin. Written informed consent was obtained from the individual(s), and minor(s)' legal guardian/next of kin, for the publication of any potentially identifiable images or data included in this article.

## Author contributions

XL, YY, QW, and ZY contributed to the study's conception and design. Material preparation and data collection were performed by YT, HW, ZL, WG, ZH, LH, and MW. Data analysis was performed by XL, YY, and ZY. The first draft of the manuscript was written by XL and YY. All authors commented on previous versions of the manuscript and read and approved the final manuscript.
